# Supplementary material for: Wood Waste from Fruit Trees: Biomolecules and Their Applications in Agri-Food Industry
Source: Biomolecules. 2022 Feb 1;12(2):238. doi: 10.3390/biom12020238 (PMC8961605; doi:10.3390/biom12020238)

Figure S1: Chemical structure of bioactive compound families investigated during this research. Own elaboration using Fisher Scientific website: https://www.fishersci.es/es/es/search/chemical/substructure.html.

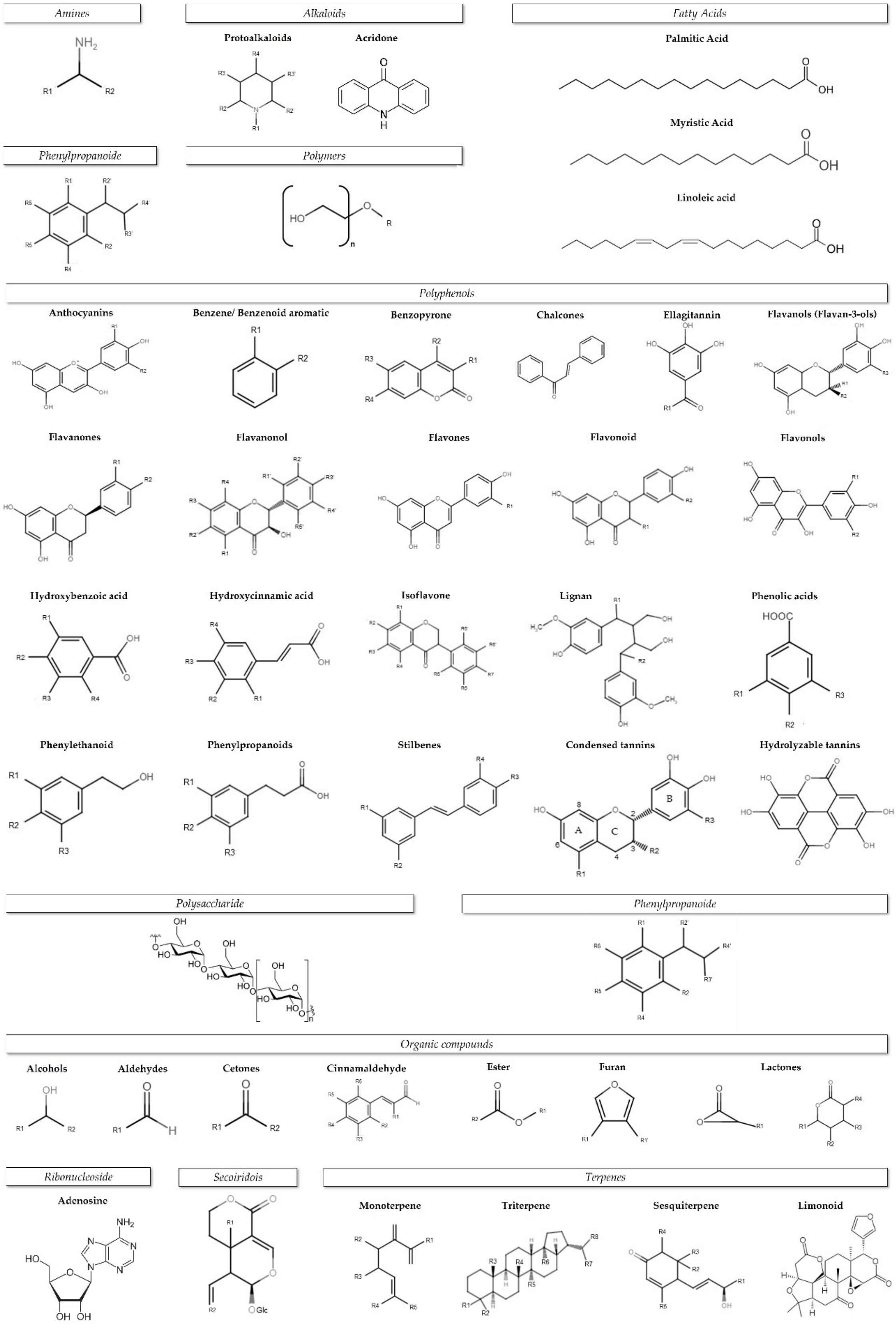

Supplement: Supplementary file 1 [file biomolecules-12-00238-s001.zip › biomolecules-1569518-SI.pdf]
